# The prominent role of percutaneous transarterial embolization in the treatment of anterior abdominal wall hematomas: the results of three high volume tertiary referral centers

**DOI:** 10.1007/s11547-023-01678-7

**Published:** 2023-07-17

**Authors:** Laura Maria Cacioppa, Chiara Floridi, Maria Adriana Cocozza, Antonio Bruno, Francesco Modestino, Claudia Martella, Marzia Rosati, Alexandro Paccapelo, Cristina Mosconi, Roberto Candelari

**Affiliations:** 1 Division of Interventional Radiology, Department of Radiological Sciences, University Politecnica Delle Marche, 60126 Ancona, Italy; 2 Department of Clinical, Special and Dental Sciences, University Politecnica Delle Marche, 60126 Ancona, Italy; 3 Department of Radiology, IRCCS Azienda Ospedaliero-Universitaria Di Bologna, Via Albertoni 15, 40138 Bologna, Italy; 4 Diagnostic and Interventional Radiology Unit, Maggiore Hospital “C. A. Pizzardi”, 40133 Bologna, Italy

**Keywords:** Conservative treatment, Embolization, Hemorrhage, Hematoma, Referral and consultation, Diagnostic imaging

## Abstract

**Purpose:**

Percutaneous transarterial embolization (PTE) represents a fast, safe and effective option for life-threatening anterior abdominal wall hematomas (AWHs) and those unresponsive to conservative treatment. Our study aims to assess cumulative results of safety, technical and clinical success of PTE performed in three high-volume tertiary referral centers and to evaluate the efficacy of the different embolic materials employed.

**Materials and methods:**

A consecutive series of 124 patients (72.8 ± 14.4 years) with AWHs of different etiology submitted to PTE were retrospectively collected and analyzed. Clinical success, defined as absence of recurrent bleeding within 96 h from PTE, was considered as primary endpoint. The results of the comparison of three groups based on embolic agent employed were also analyzed.

**Results:**

Spontaneous AWHs accounted for 62.1%, iatrogenic for 21.8% and post-traumatic for 16.1% of cases. SARS-CoV-19 infection was present in 22.6% of patients. The most commonly embolized vessels were epigastric inferior artery (*n* = 127) and superior epigastric artery (*n* = 25). Technical and clinical success were 97.6 and 87.1%, respectively. Angiographic signs of active bleeding were detected in 85.5% of cases. Four (4%) major complications were reported. The comparison of the three groups of embolic agents (mechanical, particulate/fluid and combined) showed no statistically significant differences in terms of clinical success. SARS-CoV-2 infection was found to be an independent factor for recurrent bleeding and poor 30-day survival.

**Conclusion:**

PTE performed with all the embolic agent employed in our centers is a safe and effective tool in the treatment of life-threatening anterior AWH of each origin.

## Introduction

Abdominal wall hematomas (AWHs) represent a relatively rare but potentially life-threatening entity due to blood extravasation at first contained by muscle groups and subsequently spreading into peritoneal or retroperitoneal cavities [1, 2]. The multiple conditions responsible for AWHs can be summarized in abdominal traumas, anticoagulation therapy and iatrogenic injuries[3]. Post-traumatic AWHs are found in approximately 9% of nonpenetrating traumas referred to emergency departments[4], while anticoagulant/antiplatelet therapies combined with hematologic disorders, microvascular atherosclerosis and multisystemic infections as COVID-19 disease are responsible for spontaneous AWHs[5].

In most cases, bleedings occurring in the upper abdominal quadrants are smaller and self-limited. Due to the absence of the posterior fascial sheath layer, hematomas occurring below the arcuate line are wider and prone to spreading[6, 7].

**Fig. 1:**
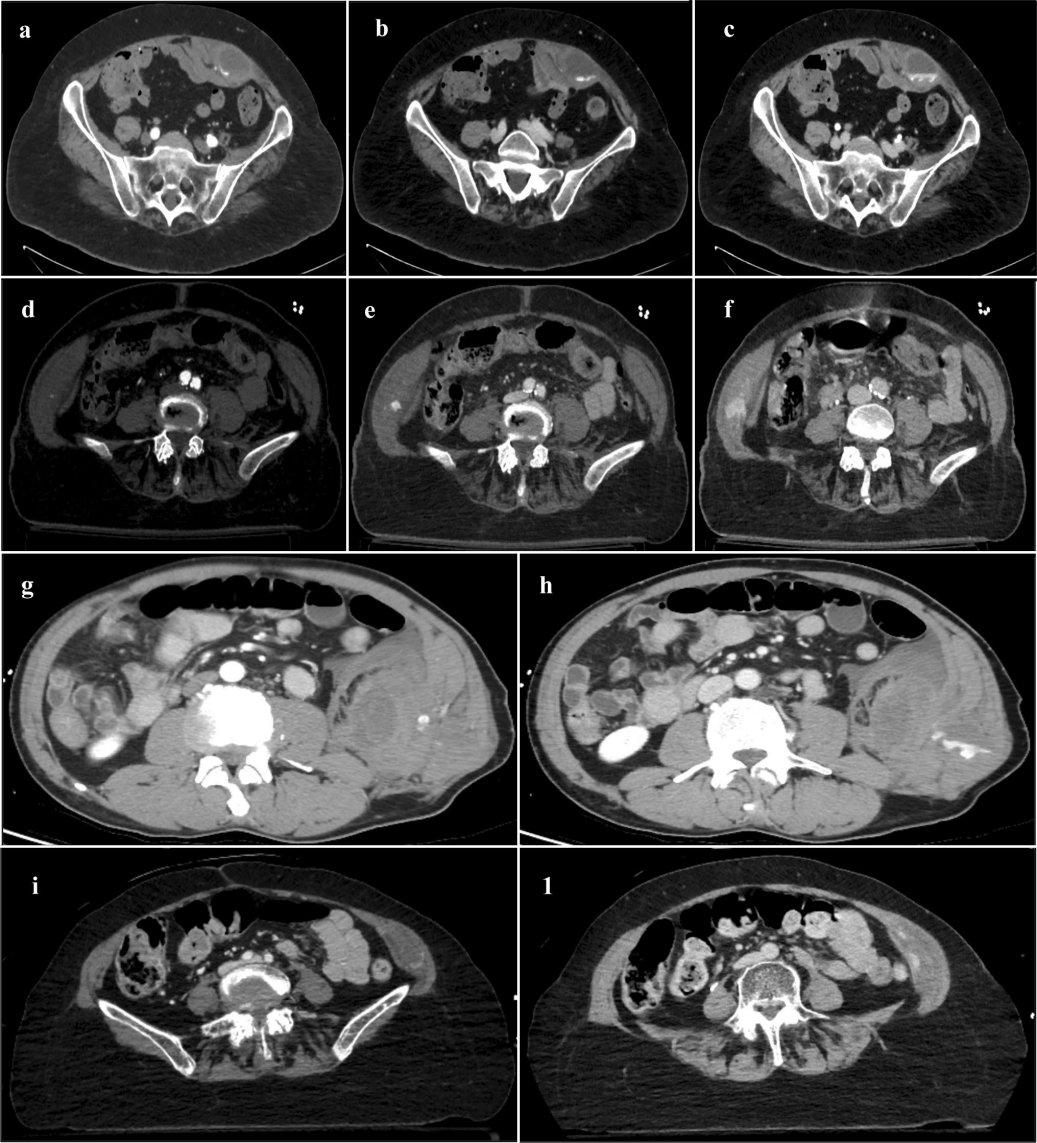
CTA images of AWHs illustrated by location. Contrast-enhanced acquisitions at arterial (**a)**, portal (**b**) and late phase (**c**) show a spontaneous AWH within the left rectus abdominis muscle with active arterial bleeding. Post-traumatic AWH of right internal oblique muscle detected at arterial (**d**), portal (**e**) and late phase (**f**). AWH arising from left external oblique muscle at arterial (**g**) and portal (**h**) phase. Post-traumatic AWH contained in left transverse muscular compartment at venous (**i**) and late (**l**) phase

The variety of clinical unspecific presentations, such as abdominal pain, anemia and hypovolemia may delay the appropriate diagnosis of AWHs. Computed tomography angiography (CTA) gives the major support in the diagnostic process providing essential information about location, characteristics and active arterial source of the AWH (Fig. 1) as well as outlining differential abdominal conditions[4].

Once diagnosed, depending on baseline, laboratory and bleeding extent conditions, the management of the AWHs may consist in conservative, endovascular or surgical treatment[8]. While hemodynamically stable patients benefit from conservative measures such as fluid blood transfusions and correction of coagulation factors, an aggressive treatment should be administered to unstable patients[9].

Nowadays, the surgical option is decreasingly being applied due to the more advanced age of patients and the high rate of failure[10, 11]. In this scenario, percutaneous transarterial embolization (PTE) represents a fast, safe and effective option for life-threatening and unresponsive to conservative treatment [2].

After digital subtraction angiography (DSA), PTE is intended to embolize the target vessel using mechanical, particulate or fluid agents[12]. If active extravasation of contrast medium cannot be seen at DSA, a blind or empiric embolization of the suspected vessel is anyway preferred[13]

Despite the great number of AWHs daily referred to the tertiary emergency departments and submitted to PTE, few studies currently report multicentric clinical and technical results as well as comparative results of the employed embolic agents.

The aim of this multicentric study was to evaluate the safety, technical and clinical success and overall survival of PTEs and to compare the efficacy of the different classes of embolic agents.

## Materials and methods

### Study population and laboratory values

A consecutive series of patients diagnosed with AWH, referred to interventional radiology departments and submitted to PTE in three tertiary referral centers were retrospectively collected and analyzed. Files and images were extracted from the three single RIS (radiology information) and PACS (picture archiving and communication) systems between January 2018 and December 2021. Clinical data and information about spontaneous, post-traumatic or iatrogenic etiology of AWHs were obtained from digital medical records. The principal risk factors for hemorrhage such as age, sex, anticoagulant medication and COVID-19 infection were also collected.

As inclusion criteria, the identification of AWH at CTA, the presence of active bleeding signs at CTA and the subsequent execution of PTE were considered, while exclusion criteria were the resolution of AWH after a surgical procedure or after conservative treatment alone.

Laboratory data such as pre- and post-interventional hemoglobin value, international normalized ratio (INR), activated partial thromboplastin time (aPTT) and platelet count were obtained from the three laboratory information systems. Pre-interventional laboratory values were measured within 12 h before the PTE and post-interventional values up to 24 h after the procedure.

The study was conducted in accordance with the Declaration of Helsinki and approved by the Institutional Review Boards. Informed consent was obtained from all subjects involved in the study.

### Pre-procedural CTA

All patients underwent multidetector-row CTA scan, all showing active extravasation of contrast medium, within a mean time of 3.4 ± 3.1 h before entering the angiography suite.

All the institutions performed the same CT standard protocol consisted of a non-contrast axial phase, followed by a bolus of 80–120 ml of iodinated low-osmolality contrast agent (300–400 mg/mL) intravenously administered by automated injection at a flow rate of 4–5 mL/s. An arterial phase was then acquired using bolus-tracking technique, placing the ROI (region of interest) at proximal abdominal aorta, followed by a venous phase (acquired with a delay of 60–70 s) and a delayed phase at 110–120 s. Coronal and sagittal reconstructions were also performed. CT images were reviewed for presence (distinct mass with a density value of 60–80 HU), location (muscular group involved) and measurement (length × width × height, cm) of the AWH, for the presence of direct signs of active bleeding (focal point-like, jet-like or rounded high density area within the AWH visualized in both arterial and venous phases), for the presence of pelvic extent and for the identification of target vessels by one radiologist with at least 5 years of experience in emergency field and one interventional radiologist in each institution, both blinded to angiographic findings.

### Procedural characteristics

All the PTE procedures were performed in the referral angiographic suite of each institution by an at least 5 years-experienced interventional radiologist and preceded by a preliminary DSA.

Patients were prepared with trichotomy, skin disinfection and sterile draping. Right or left transfemoral access was obtained, and a 4–5 Fr introducer sheath was then introduced. Angiographic overviews were obtained by automated contrast agent injection placing 4–5 Fr selective catheters, mostly Cobra-2, Pigtail and Simmons-1 (Cordis, Santa Clara, CA, USA), in abdominal aorta or more selectively in the most likely bleeding arterial branches. The involved first-to-third-order vessels were then coaxially catheterized using 1.9–2.7 F microcatheter systems, mostly Progreat (Terumo Medical, Tokyo, Japan) and Carnelian (Tokai Medical, Kasugai, Japan), conducted as close as reachable to the bleeding site.

PTE was performed both in case of angiographic direct (contrast blush) or indirect (vessel cutoff sign) findings suggestive of active bleeding or pseudoaneurysm and in case of absent or intermittent angiographic signs in an empirical CTA-guided way.

The embolic mechanical, particulate or fluid material was chosen by the discretion of the interventionalist based on arterial anatomy, extent of bleeding, patient’s blood clotting activity, risk of non-targeted embolization and his own confidence and experience. The embolic agents used in our series were: 0.018'' platinum microcoils (deployed diameter 2–6 mm), both pushable, (Boston scientific, Natick, Massachusetts) and detachable (Azur Hydrocoils, Terumo Medical, Tokyo, Japan) as mechanical agents; Gelatin sponge administered in “slurry” preparations (Spongostan®, Johnson & Johnson Medical NV, NJ,USA) and Contour® Polyvinyl alcohol (PVA microparticles (250–355 µm, Boston Scientific, Natick, Massachusetts) as particulate agents; Glubran 2® N-butyl-cyanoacrylate glue (GEM, Viareggio, Italy), administered in a 1:4 dilution ratio with ethiodized oil, as fluid agent. A combination of microcoils and Gelatin sponge was also considered.

A superselective catheterization of the adjacent and collateral vessels around the bleeding site was also performed.

## Definitions and follow-up

We defined technical success is the complete embolization of the target vessel.

Clinical success was defined as the presence of hemodynamic (blood pressure > 90 mmHg or a responsive to fluid resuscitation) and laboratory stability after the procedure. Clinical failure was considered in patients who had signs of rebleeding with the need of re-embolization within 96 h after PTE.

Targeted embolization was considered a procedure performed in the presence of direct or indirect signs of active bleeding on DSA. Otherwise, empirical embolization was intended as a PTE without angiographic signs but under the guidance of CTA findings.

Procedure-related complications were defined as suggested by the Society of Interventional Radiology [14]. Overall survival (OS) at 30 days after PTE was evaluated by digital medical records or by telephone interview if data were missing.

## Statistical analysis

All statistical analyses were conducted by SPSS version 22 software (IBM, Armonk, New York). *P* < 0.005 were considered statistically significant.

Characteristics of the study population were reported as the mean, range and median. Quantitative variables were reported both as mean ± standard deviation and median and range, as applicable. Categorical variables were reported as numbers and percentages.

Clinical success was considered as primary endpoint. OS at 30 days, technical success and complications were evaluated as secondary endpoints.

The results of univariate analysis were tested for significance using Chi-square test. Multivariate analysis was performed using binary logistic regression. Factors with *p* < 0.05 at univariate analysis were included in multivariate analysis.

## Results

### Demographical, clinical and laboratory findings

Over a total of 124 patients included, 72 (58.1%) were females, and 52 (41.9%) were males. The mean age was 72.8 ± 14.4 years. The patients enrolled complained of a spontaneous AWH in 77 (62.1%) cases, iatrogenic in 27(21.8%) of cases and post-traumatic in 20 (16.1%) of cases. In most cases, the iatrogenic bleeding occurred after major surgery: 14(11.3%) of cases, most commonly after abdominoplasty (4 cases; 3.2%) and after caesarean Sect. (3 cases;2.4%). The second most common iatrogenic cause was abdominal drainage placement, which was involved in 8(6.4%) cases.

Abdominal pain was the most frequent onset symptom (86.3%), followed by palpable mass (47.6%) and nausea (25%). Forty-three (34.7%) patients suffered of hemodynamic instability, defined as a persistent mean systolic blood pressure < 90 mmHg despite the infusion of 2 L of intravenous crystalloids or 2 units of blood products.

Principal characteristics and bleeding risk factors of study population are summarized in Table 1.

**Table 1:** Baseline characteristics, comorbidities, bleeding risk factors, types of anticoagulant therapy and laboratory values of study population (n = 124)

*Demographic*	
Age (yrs) Range	72.8 ± 14.4 (21–97)
Sex (male/female)	52(41.9%)/72(58.1%)
*Etiological*	*n (%)*
Post-traumatic Major trauma Pelvic fracture Penetrating trauma Others	20 (16.1%) 7 (5.6%) 6 (4.8%) 3 (2.4%) 3 (2.4%)
Iatrogenic Drainage placement Major surgery Abdominoplasty Caesarian section Others (pancreaticoduodenectomy, renal transplant, bilateral hysteroannessiectomy, cholecystectomy) Others	27 (21.8%) 8 (6.4%) 14 (11.3%) 4 (3.2%) 3 (2.4%) 7 (5.6%) 5 (4%)
Spontaneous	77 (62.1%)
*Clinical*	
Abdominal pain Palpable mass Nausea and vomiting Haemodynamic instability*	107 (86.3%) 59 (47.6%) 31 (25%) 43 (34.7%)
*Bleeding risk factor*	*n *(%)
Arterial hypertension Coronary heart disease Arrhythmias Kidney failure Hepatopathy2COPD** Peripheral vascular disease Malignancies SARS-CoV-2 infection	80 (64.5%) 27 (21.8%) 31 (25%) 31 (25%) 12 (9.7%) 23 (18.5%) 43 (34.7%) 34 (27.4%) 28 (22.6%)
*Anticoagulant therapy*	*n *(%)
*Ongoing therapy*	89 (71.8%)
Vitamin K Antagonists (VKAs or OAT)	21 (16.9%)
New oral anticoagulants (NOA)	18 (14.5%)
Unfractionated heparin (UFH)	5 (4%)
Low-molecular-weight heparins (LMWHs)	45 (36.3%)
*No anticoagulant therapy*	35 (28.2%)
*Laboratory value*	*Mean* ± *SD *(*range*)
Pre-procedural INR*(sec)	1.5 ± 0.9 (0.8–10.0)
Pre-procedural sPTT**(sec)	1.3 ± 0.5 (0.7–3.4)
Pre-procedural platelet count (*10^9/L)	214 ± 120 (31–674)
Pre-procedural hemoglobin value (g/dl)	8.7 ± 1.5 (5.0–15.9)
Post-procedural hemoglobin value (g/dl)	10.1 ± 1.4 (7.2–15.6)

^*^ persistent mean systolic blood pressure < 90 mmHg despite the infusion of 2 L of intravenous fluids or 2 units of blood products

^**^Chronic obstructive pulmonary disease

^***^ International normalized ratio

^****^ Activated partial thromboplastin time

A total of 89 (71.8%) patients received anticoagulant therapy: 16.9% treated by Vitamin K antagonists (OAT), 14.5% by new oral anticoagulants (NOA), 36.3% by low molecular weight heparins (LMWHs) and 4% by unfractionated heparin (UFH). SARS-CoV-19 infection was present in 28(22.6%) patients. The main indications to anticoagulation were atrial fibrillation, deep venous thrombosis and heart valves replacement.

The mean pre-procedural hemoglobin value in our population was 8.7 ± 1.5 g/dl, while post procedural hemoglobin value was 10.1 ± 1.4. Preprocedural INR and aPTT values were 1.5 ± 0.9 and 1.3 ± 0.5 s, respectively. Mean pre-procedural platelets count was 214 ± 120 10^9/L. RBC transfusions of a mean value of 7 blood units (range of 2–17) were necessary to prevent blood loss. Anticoagulant therapy and laboratory characteristics are reported in Table 1.

### Diagnostic and procedural findings

Mean AWH volume measured at CTA was 1063 cm^3^. The AWHs involved in 91.1% of cases the rectus abdominis muscle, in association with internal oblique muscle in 29 (23.4%) cases and with transversus abdominis muscle in 31 (25%) cases. The right abdominal site was involved in 46(37.1%) of cases. In 14(11.3%) cases, the AWH spread bilaterally and in 64(51.6%) had a pelvic extension. Due to the large size of hematoma, two of the latter cases complained of dislocation and compression of pelvic structures resulting in hydroureteronephrosis, one for bilateral ureteral compression and one for bladder compression.

Direct or indirect signs of active bleeding were assessed at DSA in 106 patients (85.5%). In 18 patients (14.5%), no angiographic evidence of active bleeding was detected. The most commonly embolized vessels were left epigastric inferior (*n* = 67), right epigastric inferior (*n* = 60), superior epigastric (*n* = 25), right deep circumflex iliac (*n* = 14), left deep circumflex iliac (*n* = 13) and gluteal arteries(*n* = 4). PTE was performed in a single arterial territory in 61 (49.2%) patients and in multiple territories in 63 (50.8%) patients.

Mechanical agents were employed in 45 (36.3%) patients, with an average number of 6.4 microcoils per patient. Particulate and fluid agents employed were gelatin sponge in 64(51.6%) patients with an average quantity of 4.4 ml per patient, N-butyl cyanoacrylate in 10(8.1%) patients (4.1 ml per patient) and PVA particles in 8(6.5%) patients. A combination of microcoils and gelatin sponge was preferred in 32(25.8%) cases. These findings are summarized in Table 2.

**Table 2:** diagnostic and procedural findings, outcomes and complications

*Bleeding site*	*n *(%)
Rectus abdominis muscle Right Left Bilateral	113 (91.1%) 46 (37.1%) 53 (42.7%) 14 (11.3%)
Internal oblique muscle Alone Combined with rectus abdominis muscle	35 (28.2%) 6 (4.8%) 29 (23.4%)
Transverse abdominal muscle Alone Combined with rectus abdominis muscle	36 (29%) 5 (4%) 31 (25%)
*Bleeding extent*	*n *(%)
Pelvis Lower limb	64 (51.6%) 4 (3.2%)
*Hematoma size* (antero-posterior^latero-lateral^cranio-caudal diameter, cm)	*Mean* ± *SD *(*range*)
*antero-posterior* (cm) *latero-lateral* (cm) *cranio-caudal* (cm) *volume* (cm^3^)	8.8 ± 3.9 (3.5–20.0) 7.7 ± 3.5 (2.0–20.0) 13.4 ± 5.4 (2.5–40.0) 1063 ± 1086 (20–7040)
*CTA-DSA interval*	*hours*
Mean ± SD Range	3.4 ± 2.9 (0.5–24.0)
*Intervention time*	*min*
Mean ± SD Range	67.6 ± 40.3 (20–320)
*DSA signs*	*n *(%)
Active bleeding (targeted embolization) No signs of active bleeding (blind embolization)	106 (85.5%) 18 (14.5%)
*Embolized artery*	*n (%)*
Left inferior epigastric artery Right inferior epigastric artery Left deep circumflex iliac artery Right deep circumflex iliac artery Internal thoracic or mammary artery Others (gluteal, obturator, hypogastric, iliolumbar, uterine arteries)	67 (54%) 60 (48.4%) 13 (10.5%) 14 (11.3%) 25 (20.2%) 16 (12.9%)
*Type of embolic agents*	*n *(%)
Mechanical only (microcoils) Particulate (Contour 150–250 µm) Liquid (Glubran) Temporary particulate (Gelatin Sponge) Combined (microcoils + gelatin sponge) None (dissection, covered stent)	45 (36.3%) 8 (6.5%) 10 (8.1%) 64 (51.6%) 32 (25.8%) 2 (1.6%)
*Quantity of embolic agent*	*Mean* ± *SD (range)*
Glubran (ml) Gelatin Sponge (ml) Number of microcoils	4.1 ± 2.2 (1–8) 4.4 ± 2.0 (2–10) 6.4 ± 3.5 (2–20)
*Outcome*	*n *(%)
Technical success	121 (97.6%)
Clinical success	108 (87.1%)
Recurrent bleeding within 96 h	16 (12.9%)
Thirty-day survival	109 (87.9%)
Major complications Abdominal compartment syndrome* Hemorrhagic shock Other documented post-procedural minor complications	5 (4%) 3 (2.4%) 2 (1.6%) 10(8.1%)

^*^pre-procedural complication due to the large size of the abdominal wall haematoma

## Survival and outcomes

Technical success was achieved in 121(97.6%) patients. One case of focal and temporary dissection of the target vessel and two cases of unable catheterization, due to extensive ostial calcifications, were recorded.

**Fig. 2:**
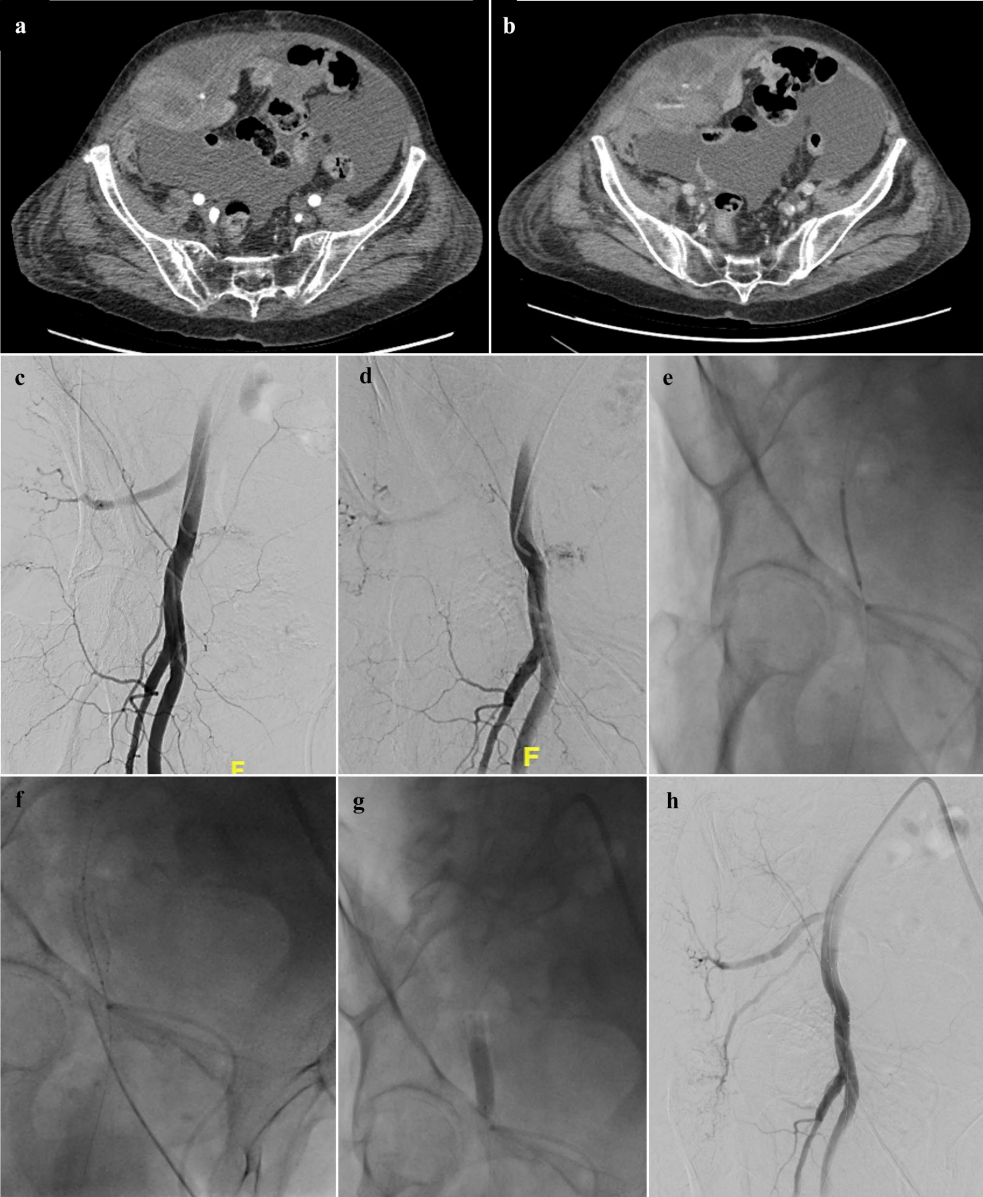
CT and DSA images of a technically failed PTE of the target vessel of a spontaneous AWH. Clinical success was anyway reached by a covered stent placement. Axial CT images after contrast-enhanced acquisition at arterial and late phase showed a large hematoma within the right rectus abdominis with active arterial bleeding (**a**–**b**). At DSA, active contrast extravasations from small multiple proximal branches of right inferior epigastric artery were detected (**c**–**d**). Unable catheterization, due to extensive ostial calcifications of right inferior epigastric artery and 8 mm covered stent placement at distal segment of right iliac external artery (**e**). Stent deployment and balloon post-dilation (**f**–**g**). At final DSA, no signs of active bleeding were detected (**h**)

Bleeding interruption was obtained by the spontaneous vascular defect in the first case and by a covered stent placement in the two others (Fig. 2). The three cases of technical failure all belong to the spontaneous bleeding group.

Clinical success was achieved in 108 (87.1%) cases. Clinical failure as 96 h recurrent bleeding after PTE was detected in 16 patients (12.9%), all of them managed with a single re-embolization. The overall 30-day survival rate was 87.9%. No statistically significant differences were found between males and females in terms of clinical success and overall survival.

Minor complications after PTE were encountered in 10 (8.1%) patients, most of them related to vascular access site. In detail, six cases of superficial femoral artery pseudoaneurysm and four cases of significant hematoma were detected. No complications related to nontarget embolization and no cases of skin ischemia related to particulate embolic materials were recorded.

As major complications, the development of abdominal compartment syndrome (2.4%) due to the large size of AWH, two of them submitted to laparotomic evacuation and one to percutaneous drainage placement and two cases of hemorrhagic shock 24 h after PTE were registered. Primary and secondary endpoints are reported in Table 2.

The results of univariate analysis of potential risk factors for recurrent bleeding within 96 h after PTE are illustrated in Table 3. At univariate analysis, SARS-CoV-19 infection (71.4% vs 91.9%, *p* = 0.02) and transverse abdominal muscle involvement (75.9% vs 92.4%, *p* = 0.04), whose arterial supply arises from lumbar, deep circumflex iliac and external iliac arteries, were identified as potential risk factors for clinical failure. A multivariable regression model further confirmed both these characteristics as independent risk factors for rebleeding (*p* = 0.029 and *p* = 0.049, respectively). Again, SARS-CoV-19 infection has proven to be an independent risk factor for poor 30-day survival: 70/74 (94.6%) vs 13/21 (61.9%), < 0.001.

**Table 3:** Univariate and multivariate analysis of risk factors for clinical failure

	Clinical Success Rate				Univariate	Multivariate
	*n* (%)				*p*-value	*p*-value
*Type of embolic agent*						
Mechanical	37	/	45	(82.2%)		
Liquid/particulate	41	/	45	(91.1%)	0.455	/
Combined	28	/	32	(87.1%)		
*Covid*						
Positive	18	/	28	(64.3%)	< 0.001	< 0.001
Negative	68	/	74	(93.8%)		
*Etiology*						
Spontaneous	65	/	77	(84.4%)		
Iatrogenic	23	/	27	(85.2%)	0.170	/
Traumatic	20	/	20	(100.0%)		
*Anticoagulant treatment*						
Yes	75	/	89	(84.3%)	0.232	/
No	33	/	35	(94.3%)		
*Rectus abdominis muscle*						
Yes	98	/	113	(86.7%)	1.000	/
No	10	/	11	(90.9%)		
*Internal oblique muscle*						
Yes	28	/	35	(80.0%)	0.148	/
No	80	/	89	(89.9%)		
*Transverse abdominal muscle*						
Yes	26	/	36	(72.2%)	0.003	0.009
No	82	/	88	(93.2%)		
*Pelvic extent*						
Yes	54	/	64	(84.4%)	0.427	/
No	54	/	60	(90.0%)		
*Lower limb extent*						
Yes	4	/	4	(100.0%)	1.000	/
No	104	/	120	(86.7%)		
*DSA*						
Positive	90	/	106	(84.9%)	0.125	/
Negative	18	/	18	(100.0%)		

The comparative results of three groups of patients divided according to embolic agent employed (mechanical versus particulate/fluid versus combined) are shown in Table 4.

**Table 4:** Comparative results of the three groups of embolic agents

	Mechanical *n* = 45				Liquid/particulate *n* = 45				Combined *n* = 32				*p*-value
Clinical success	37	/	45	(82.2%)	41	/	45	(91.1%)	28	/	32	(87.5%)	0.455
30-day survival	37	/	45	(82.2%)	43	/	45	(95.6%)	28	/	32	(87.5%)	0.137
Complications	2	/	45	(4.4%)	1	/	45	(2.2%)	2	/	32	(6.3%)	0.672
Covid +	9	/	45	(20.0%)	9	/	45	(20.0%)	10	/	32	(31.3%)	0.430
Etiology													
*Spontaneous*	30	/	45	(66.7%)	27	/	45	(60.0%)	18	/	32	(56.3%)	
*Iatrogenic*	6	/	45	(13.3%)	11	/	45	(24.4%)	10	/	32	(31.3%)	0.418
*Traumatic*	9	/	45	(20.0%)	7	/	45	(15.6%)	4	/	32	(12.5%)	
Anticoagulant treatment	31	/	45	(68.9%)	34	/	45	(75.6%)	22	/	32	(68.8%)	0.731
Rectus abdominis muscle	40	/	45	(88.9%)	41	/	45	(91.1%)	30	/	32	(93.8%)	0.763
Internal oblique muscle	17	/	45	(37.8%)	6	/	45	(13.3%)	12	/	32	(37.5%)	0.016
Transverse muscle	18	/	45	(40.0%)	6	/	45	(13.3%)	12	/	32	(37.5%)	0.011
Pelvis extent	24	/	45	(53.3%)	18	/	45	(40.0%)	22	/	32	(68.8%)	0.045
Lower limb extent	1	/	45	(2.2%)	1	/	45	(2.2%)	2	/	32	(6.3%)	0.547
Positive DSA	43	/	45	(95.6%)	36	/	45	(80.0%)	26	/	32	(81.3%)	0.068

The comparison between the three groups, similar in terms of number of patients, did not show statistically significant differences in terms of clinical success, OS and complication rates.

## Discussion

Anterior AWH is a relatively rare condition, whose precise incidence is still unclear due to the lack of focused studies.

Rectus sheath hematoma was previously identified as the cause of the 1.8% of acute-onset abdominal pain, an incidence slightly higher than posterior wall hematoma[12]. To date, our series is one of the largest conducted with a multicentric design and focused on AWH only arised within anterior compartment.

Previous anatomical studies identified anterior lower quadrants as a preferential target for both spontaneous and iatrogenic bleedings[15], due to the marked weakness of vessel wall of inferior epigastric artery and to the anatomical susceptibility of rectus abdominis to tensional stresses [15, 16].

Advanced age has been largely confirmed as an important risk factor for AWHs, especially if associated with multiple comorbidities[17]. According to our mean age of 72.8 years, the studies of Barral et al. and Di Pietro et al., reported a mean age of 72 ± 14 years and 71 ± 12 years, respectively [12, 18]. Furthermore, the predominance of female sex (58.1%) of our series is consistent with previous findings and confirms women’s susceptibility to AWH due to the lower muscular mass[15].

Regarding etiology, previous studies reported iatrogenic manipulations as the most common cause and in particular paracentesis in patients with chronic liver disease, subcutaneous injections and catheter insertions[3, 9]. Conversely, in our series, AWHs requiring PTE occurred more frequently spontaneously.

The role of CTA in the identification of bleeding signs and in embolization planning was widely recognized, with the highest detection rate of active bleeding reported of 93%[10, 19–21], while data on the detection rate of active bleeding on DSA have been less frequently reported. Our results about DSA bleeding detection are aligned and with those reported by Di Pietro et al. and Touma et al., 79 and 85%, respectively[12, 13]. Blind embolization was in general rarely performed in our series compared to targeted embolization (85.5% vs. 14.5% of cases), and this technique showed similar outcomes to non-empiric approach. The number of non-targeted embolizations was also lower than other previous series, suggesting a more conservative strategy in smaller and self-limiting AWHs and an accurate and selective catheterization of each suspected vessels.

The results obtained in terms of clinical success as absence of rebleeding are encouraging and higher than those reported in last series[12, 13], while technical success rate of 97.6% was aligned to previous results. Regarding OS, our results reported a 30-day survival of 87.9% compared to recently reported OS rates of 77.3, 75.1 and 70%[1, 13, 22].

**Fig. 3:**
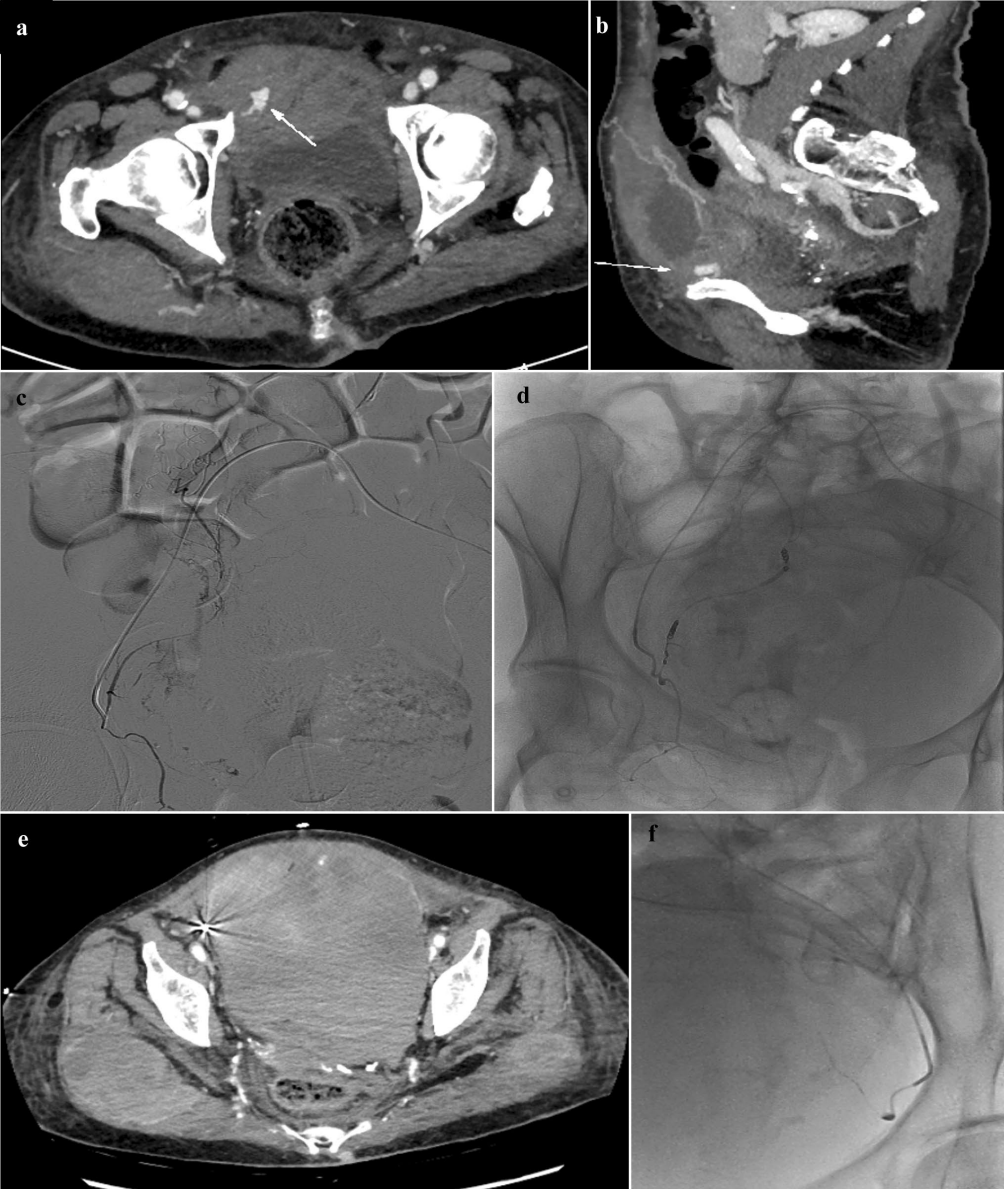
CT and DSA images of a case of recurrent spontaneous AWH in a SARS-CoV-19 patient. Axial (**a**) and sagittal (**b**) CT images after contrast-enhanced acquisition at arterial phase showed a large hematoma within the right rectus abdominis with active arterial bleeding. At DSA, multiple contrast extravasations from right inferior epigastric artery were detected (**c**). PTE procedure was performed with “front and back” technique using 2 and 3-mm microcoils (**d**). Twenty-four hours after PTE, a recurrent AWH with pelvic spreading was detected at contrast-enhanced CT (**e**). At DSA, the left inferior epigastric artery showed active extravasation and embolized using 2-mm microcoils (**f**)

Interestingly, SARS-CoV-2 infection was identified as an independent risk factor for recurrent bleeding and low 30-day survival rate (Fig. 3). Spontaneous bleedings as severe complications of the anticoagulant therapy with LMWH recommended to reduce thrombotic risk in critically ill SARS-CoV-2 patients have been widely described in the literature (23,24). Cytokine release syndrome, hypoxia and immobilization in patients with SARS-CoV-2 infection were identified as being responsible for the hypercoagulable state resulting in fatal outcomes and requiring anticoagulation use[25].

Nevertheless, few, limited and single-center series reported clinical success and overall survival of PTEs performed in SARS-CoV-2-affected people[23–26]. Among previous studies, none considered SARS-CoV-2 infection as an independent clinical factor potentially responsible for AWH and in a consecutive series exclusively focused on anterior compartment.

Due to the increasingly use of PTE for persisting AWHs after conservative treatment and to the growing preference of PTE over surgical interventions, further investigations on procedural issues and on embolic agents are necessary[2, 8, 27, 28]. Given the heterogeneity of reported data, at present time no clear evidence to recommend one embolic agent over another exists[13, 29, 30]. Furthermore, comparing different embolic materials may be challenging due to the multiple factors determining the choice, such as the preference of the interventionalist, anatomy, caliber and tortuosity of the target vessel, general and coagulative patient characteristics and, no less importantly, the ready availability of materials in angiographic suits[29, 30].

In our study, these different factors resulted sufficiently homogenized due to the similarities of our three institutions, the standards-based procedures, the similar education of interventional radiologists in embolic properties and the same availability of materials of the three hubs.

Based on these considerations, we were able to establish three groups of embolic agents with analog properties. The results obtained from this comparative analysis did not demonstrate statistically significant differences in terms of clinical success, OS and complication rates and in particular no differences between mechanical and liquid/particulate agents were noted in terms of 96-h rebleeding, even in presence of anticoagulant treatment or SARS-CoV-2 infection. Our data are inconsistent with previous results, which sustained the use of glue as a first choice on account of its ability to promote permanent occlusion in patient with deranged coagulation and distal embolization in cases of small tortuous vessels and rich collateral nets[2, 15, 31]. Microcoils alone or in combination with other agents may be ideal for embolization of single or few vessels of large diameter, as is the case of anterior AWHs, avoiding non-target embolization and skin necrosis[9]. Moreover, in our institutions, microcoils placement is performed as distally as possible in case of multiple collaterals, often associated with a particulate agent as gelatin sponge.

In the present series, 4% of patients developed major complications, 75% of whom represented by abdominal compartment syndrome. Despite its low incidence, this sequela may be totally avoided performing PTE as an early procedure in patients with initial large-volume AWHs [22, 32]. No major complications directly related to PTE procedure were observed, and notably no cases of non-targeted embolization were documented.

Despite the present study is among the largest in this area, several and important limitations stem from its retrospective design with the lack of a control population managed conservatively or surgically. Some important differences in terms of patient selection and post-procedural management from center to center should also be considered.

The record of complications, relatively underestimated by lacks in medical documentation or missed correlation to PTE, and the limited follow-up make firm conclusions difficult to extract. Furthermore,preprocedural INR and aPTT values may be affected by an under- or over-anticoagulated patient status Their future correlation with hematoma size, the need for embolization and transfusions and the average number of transfused units is auspicable.

The obtained results confirm safety and efficacy of PTE in the treatment of AWHs of each etiology. These conclusions may be also applied in the absence of angiographic proofs of bleeding if an AWH was documented at CTA. PTE procedures performed using the three major classes of embolic materials have demonstrated to be equally feasible, safe and efficient in bleeding control.
